# Trending Longitudinal Agreement between Parent and Child Perceptions of Quality of Life for Pediatric Palliative Care Patients

**DOI:** 10.3390/children4080065

**Published:** 2017-08-01

**Authors:** Meaghann S. Weaver, Cheryl Darnall, Sue Bace, Catherine Vail, Andrew MacFadyen, Christopher Wichman

**Affiliations:** 1 Children’s Hospital and Medical Center Omaha, Division of Palliative Care, 8200 Dodge Street, Omaha, NE 68114, USA; meahcloud@yahoo.com (C.D.); sbace@childrensomaha.org (S.B.); cvail@childrensomaha.org (C.V.); amacfadyen@childrensomaha.org (A.M.); 2 University of Nebraska Medical Center, Department of Biostatistics, Omaha, NE 68198, USA; Wichmchristopher.wichman@unmc.edu

**Keywords:** quality of life, pediatric palliative care, patient reported outcomes

## Abstract

Pediatric palliative care studies often rely on proxy-reported instead of direct child-reported quality of life metrics. The purpose of this study was to longitudinally evaluate quality of life for pediatric patients receiving palliative care consultations and to compare patient-reported quality of life with parent perception of the child’s quality of life across wellness domains. The 23-item PedsQL™ V4.0 Measurement Model was utilized for ten child and parent dyads at time of initial palliative care consultation, Month 6, and Month 12 to assess for physical, emotional, social, and cognitive dimensions of quality of life as reported independently by the child and by the parent for the child. Findings were analyzed using Bland–Altman plots to compare observed differences to limits of agreement. This study revealed overall consistency between parent- and child-reported quality of life across domains. Physical health was noted to be in closest agreement. At the time of initial palliative care consult, children collectively scored their social quality of life higher than parental perception of the child’s social quality of life; whereas, emotional and cognitive quality of life domains were scored lower by children than by the parental report. At the one year survey time point, the physical, emotional, and social domains trended toward more positive patient perception than proxy perception with congruence between quality of life scores for the cognitive domain. Findings reveal the importance of eliciting a child report in addition to a parent report when measuring and longitudinally trending perceptions on quality of life.

## 1. Introduction

The vast majority of symptom burden and quality of life metrics from children receiving palliative care are obtained from a proxy report rather than the voice of the child. In a systematic review of research papers measuring outcomes for children with cancer, only four out of 26 papers (15.4%) included actual patient-reported outcomes while six (23.1%) included parent-reported outcomes, and five (19.2%) included nursing reported outcomes [1]. In another review of pediatric palliative care peer-reviewed publications specific to child outcomes, only nine out of 72 (13%) papers provided direct patient perspectives [2]. Reasons for exclusion of the child’s voice in quality of life (QOL) reporting includes that children may be unwilling or unable to respond for themselves due to developmental stage or illness impact or because of cognitive impairment. The reason may also be that providers and researchers are not routinely pursuing direct child-reported metrics as part of the gold standard approach to care and research [3]. Studies coordinating caregiver and pediatric child quality of life metrics in pediatric palliative care are meaningful but few [4].

It cannot be assumed that clinician or even parent report accurately reflects the burden of illness or treatments as perceived by the child. A growing body of adult literature reminds clinicians that the clinician report of symptoms relevant to overall quality of life systematically under-reports both the prevalence and the severity of these symptoms [5,6]. Comparisons of a child report versus a parental proxy report of symptoms has revealed varied levels of agreement with higher correlation for observable symptoms such as nausea (the observation being presumed change in eating pattern or even emesis) and pain (the observation being activity level or nonverbal/verbal signs of discomfort) and poorer level agreement for less observable symptom profiles [7,8]. Agreement between the child report and parent report of symptom burden varies with child age, with poorer agreement for adolescent patients than for younger children [9,10]. Agreement is further confounded by a parent’s personal physical and mental health status impacting parental perception of his/her own child’s quality of life [11].

The translation of symptom burden into perceived quality of life warrants a direct patient report with supplemental, informative parental insight to place the child’s experience into the family interpretation of the child’s experience.

## 2. Methods

### 2.1. Patients

This prospective study followed a total of ten patients (four males and six females) aged 5 years through 18 years (mean 12.4 years) from time of initial palliative care consultation through one full year of palliative care integrated service. Only ten out of 87 total consulted patients were able to provide patient voice due to medical fragility (neuro-cognitive participatory or development level). Of these ten participants, three utilized the 5–7-year-old tool; three used the 8–12-year old tool; and four completed the 13–18-year old tool based on age at time of study participation. Primary diagnoses included four children with neurodegenerative conditions, three with cardiac conditions, two with pulmonary conditions, and one with a genetic condition. Two families completed the quality of life scales in Spanish, all others were completed in English.

### 2.2. Study Design

This study was approved as a Quality Improvement Process Study by the Institutional Review Board. Child and parent demographic information was collected at baseline. QOL questionnaires were administered at baseline (defined as time of initial palliative care consultation), Month 6, and Month 12 of integrated palliative care services. Integrated palliative care services meant longitudinal inclusion of a palliative care team and palliative care case management with established needs assessment/goals of care guiding interventions across outpatient and inpatient medical settings. The questionnaire was completed separately by each participating child and parent during scheduled palliative care visits either as an inpatient or outpatient. Each child had the option for the questionnaire items to be read out loud if the child preferred audible scale administration.

### 2.3. Measures

Quality of life was assessed using the Pediatric Quality of Life Inventory (PedsQL™ Copyright © 1998 JW Varni, Ph.D. All rights reserved), a 23-item Likert-type scale measuring: physical, social, cognitive, and emotional domains [12]. The validated pediatric scale has parallel instruments for child and parent administration [13]. The Likert scale scores are converted to a 0-, 25-, 50-, and 100-point scale, with higher scores reflecting a higher perceived quality of life for that domain.

### 2.4. Statistical Analysis for Parent–Child Quality of Life Agreement

**Figure 1:**
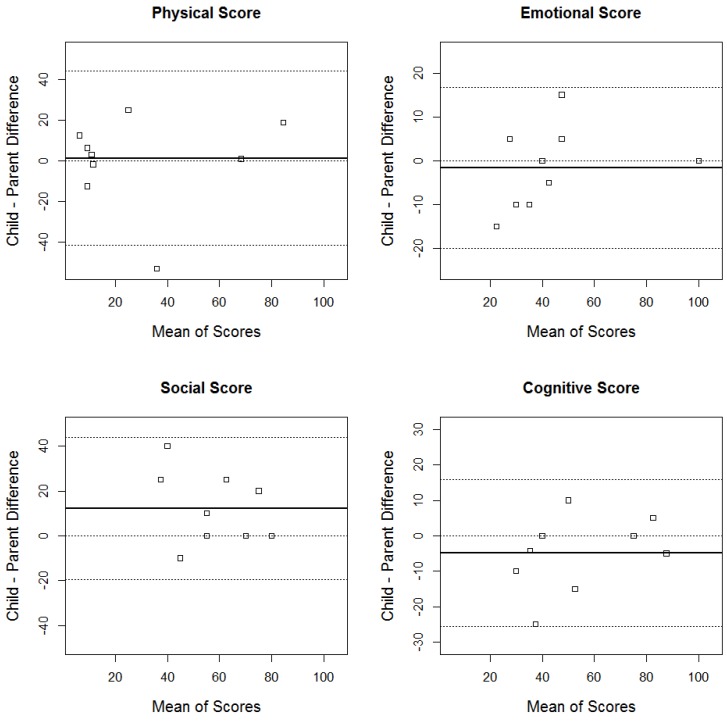
Bland–Altman agreement plots for the four domains at baseline. The solid black line represents the mean of the child–parent differences. The two extreme dotted gray lines mark the upper and lower agreement boundaries.

Bland–Altman plots and absolute agreement (scatter) plots were used to assess the agreement between parent and child when scoring the QOL questionnaire on the Physical, Social, Cognitive, and Emotional Scales [14]. Bland-Altman plots are used to compare observed differences to limits of agreement which are based on the mean of all differences +/− z1 − α2 times the standard deviation of the differences. The Bland–Altman plot offers visual appeal since the solid black horizontal line represents the average of the difference between scores across all child–parent combinations. This graphical format enables convenient visualization of collective analyses of quality of life agreement between patient–proxy reports (Figure 1). The outlier marks then revealed moments in which a parent or child differed significantly in perception of quality of life.

## 3. Results

At baseline, Month 6, and Month 12 there were 10, 6, and 5 parent–child pairings that scored the QOL assessment, respectively. Three of these parent–child pairings scored the assessment at all three time points. Reason for loss of dyad-based date was death in four cases and loss of ability for child to interact in the other cases (intubated, sedate, or decline in conversational ability).

Figure 1 reveals overall good agreement between parent and child across domains at time of initial palliative care consultation. Agreement was judged as good based on 10 out of 10 child–parent differences lying within the agreement boundaries for the emotional, social and cognitive domains and 9 out of 10 lying within the agreement boundaries for the physical domain. For physical health quality of life domains, the parent and child report on quality of life were not far off zero at baseline (the average difference between child and parent scores was 1.2, revealing agreement between patient and proxy report). However, for the social domain the child tended to score himself/herself higher than parental perception of social quality of life by an average of 12.2. For emotional domain and cognitive interaction, the children collectively scored themselves lower than parental perception of quality of life in these domains, 1.7 and 4.9 on average, respectively.

At six months, the parent–child assessment of physical and social quality of life measures were consistent between parent–child with children still scoring their own emotional and cognitive domains lower than the parental report. At a year, the children began to score their physical, emotional, and social domains collectively higher than parental perception.

## 4. Discussion

Inquiring into quality of life fosters insight into the effect of disease trajectory on a child and overall perception of lived experience. This novel study serves as a pilot model of feasibility which could next be utilized in an earlier palliative integration model for larger and longer data points. Although there is an increasing call for pediatric providers to foster patient voice, pediatric care teams often grade symptoms and report quality of life or for the parent to report such as proxy. The graded symptoms which lead to patient-interpretation of quality of life often include subjective symptoms such as nausea, dyspnea, fatigue, insomnia, anxiety, depression, and pain. Eliciting a patient report not just for symptom report but for collective translation into perceived quality of life fosters the overall child perception of lived experience.

The finding that parents and children had the closest agreement in physical quality of life assessment may hint that the physical symptomatology is more objectively measured based on observed behavior changes or biomedical changes. Parent trend toward over-elevating a child’s emotional and cognitive wellness at time of diagnosis may imply that children are experiencing more psychosocial and mental toll than is readily recognized within families. This speaks to opportunity for palliative care teams to proactively screen for total pain dimensions and not just physical evaluations in pediatric palliative care intake.

The finding that children viewed their social wellness as higher than parental perception may be a reflection of generational differences regarding the perceived relational aspects of social media or peer support. We observe many pre-teens and adolescent children who text or video-engage with peers as still feeling socially supported/connected with these friendships, while parents may perceive less in-person engagement in playdates as social alienation.

We were fascinated to note the trend of decreased agreement between child–parent quality of life report over time with children trending toward self-reporting higher total quality of life than parent perception. This may be an area of future inquiry, particularly to study whether ego-resiliency may help patients adapt to the changes associated with the illness [15]. This raises thoughts as to how this trend may differ for patients who are not followed by a palliative care team.

A limitation of the study is the lack of direct questioning on spiritual dimensions of wellness in the instrument, as we recognize that spirituality and sense of meaning weigh heavily on quality of life perceptions. Our study was further limited by small sample size, although the progressive morbidity due to disease progression (loosing ability to self-report) and eventual mortality are inevitable in our study sampling. Loss of participants longitudinally is a reality when caring for a pediatric palliative care population toward natural end of life. While it is possible to monitor scores over twelve months, the large reduction in the number of patients who were able to complete quality of life scores represents a reality of our patient population.

Our clinical team adapts this plot approach to guide our understanding of collective quality of life trends for children receiving palliative care consultations. This plot approach enables monitoring for congruence or discongruence between child and proxy perspectives. Quality of life metrics foster a meaningful opportunity to honor patient voice, while also attending to family interpretation of child experience.

## 5. Disclosures

This research did not receive any specific grant from funding agencies in the public, commercial, or not-for-profit sectors.

## References

[1] Hinds P.S., Brandon J., Allen C. (2007). Patient-reported outcomes in end-of-life research in pediatric oncology. J. Pediatr. Psychol..

[2] Weaver M.S., Heinze K.E., Bell C.J., Heinze K.E., Bell C.J., Wiener L., Garee A.M., Kelly K.P., Casey R.L., Watson A. (2016). Establishing psychosocial palliative care standards for children and adolescents with cancer and their families: An integrative review. Palliat. Med..

[3] Cremeens J., Eiser C., Blades M. (2006). Factors influencing agreement between child self-report and parent proxy-reports on the Pediatric Quality of life Inventory 4.0 (PedsQL™) generic core scales. Health Qual. Life Outcomes.

[4] Rosenberg A.R., Oreliana L., Ullrich C., Kang T., Geyer J.R., Feudtner C., Dussel V., Wolfe J. (2016). Quality of life in children with advanced cancer: A Report from the PediQUEST Study. J. Pain Symptom Manag..

[5] Fromme E.K., Eilers K.M., Mori M., Hsieh Y.C., Beer T.M. (2004). How accurate is clinician reporting of chemotherapy adverse effects? A comparison with patient-reported symptoms from the Quality-of-Life Questionnaire C30. J. Clin. Oncol..

[6] Basch E. (2010). The missing voice of patients in drug-safety reporting. N. Engl. J. Med..

[7] Eiser C., Morse R. (2001). Can parents rate their child’s health-related quality of life? Results of a systematic review. Qual. Life Res..

[8] Collins J.J., Devine T.D., Dick G.S., Johnson E.A., Kilham H.A., Pinkerton C.R., Stevens M.M., Thaler H.T., Portenoy R.K. (2002). The measurement of symptoms in young children with cancer: The validation of the Memorial Symptom Assessment Scale in children aged 7–12. J. Pain Symptom Manag..

[9] Chang P.C., Yeh C.H. (2005). Agreement between child self-report and parent proxy-report to evaluate quality of life in children with cancer. Psycho-Oncology.

[10] Waters E., Stewart-Brown S., Fitzpatrick R. (2003). Agreement between adolescent self-report and parent reports of health and well-being: Results of an epidemiological study. Child Care Health Dev..

[11] Panepinto J.A., Hoffmann R.G., Pajewski N.M. (2010). The effect of parental mental health on proxy reports of health-related qualify of life in children with sickle cell disease. Pediatr. Blood Cancer.

[12] Varni J.W., Seid M., Rode C.A. (1999). The PedsQL™: Measurement Model for the Pediatric Quality of Life Inventory. Med. Care.

[13] Varni J.W., Burwinkle T.M., Seid M., Skarr D. (2003). The PedsQL™ 4.0 as a pediatric population health measure: Feasibility, reliability, and validity. Ambul. Pediatr..

[14] Bland J.M., Altman D.G. (1999). Measuring agreement in method of comparison studies. Stat. Methods Med. Res..

[15] Mandrell B., Baker J., Levine D., Gattuso J., West N., Sykes A., Gajjar A., Broniscer A. (2016). Children with minimal chance for cure: Parent proxy of the child’s health-related quality of life and the effect on parental physical and mental health during treatment. J. Neurooncol..

